# Temporal and spatial variability in snow cover over the Xinjiang Uygur Autonomous Region, China, from 2001 to 2015

**DOI:** 10.7717/peerj.8861

**Published:** 2020-04-08

**Authors:** Wenqian Chen, Jianli Ding, Jingzhe Wang, Junyong Zhang, Zhe Zhang

**Affiliations:** 1 Key Laboratory of Smart City and Environment Modelling of Higher Education Institute, College of Resources and Environment Sciences, Xinjiang University, Urumqi, Xinjiang, China; 2 Key Laboratory of Oasis Ecology, Xinjiang University, Urumqi, Xinjiang, China; 3 College of Chemistry and Environmental Engineering, Shenzhen University, Shenzhen, Guangdong, China; 4 Key Laboratory for Geo-Environmental Monitoring of Coastal Zone of the Ministry of Natural Resources & Guangdong Key Laboratory of Urban Informatics & Shenzhen Key Laboratory of Spatial Smart Sensing and Services, Shenzhen University, Shenzhen, Guangdong, China

**Keywords:** Arid regions of Central Asia, Xinjiang, MOD10A2, Seasonal snow cover, NSACI index, Temporal and spatial variation

## Abstract

Xinjiang, China, is a typical arid and semi-arid region of Central Asia that significantly lacks freshwater resources, and the surface runoff in this region is mainly supplied by mountain glacier and snow cover meltwater. Based on the above background and issues of transnational water resources between Xinjiang and Central Asia along the Silk Road Economic Belt, which were highlighted in the major strategy of “The Belt and Road”, this study analysed the spatial and temporal variations in snow cover and snow cover days in the Xinjiang region from 2001 to 2015. The study area includes four subregions: Northern Xinjiang, Southern Xinjiang, Eastern Xinjiang and the Ili River Valley. Moderate-resolution Imaging Spectroradiometer (MODIS) 8-day snow cover data were used after removing clouds by combining MOD10A2 and MYD10A2. The results showed that seasonal snow cover occurred from October to April in most regions of Xinjiang and that this snow cover consisted of two processes: snow accumulation and snow ablation. The maximum snow cover occurred in January, whereas the minimum snow cover occurred from July to August. During the seasonal snow cover period, the snowfall rates in Northern Xinjiang and the Ili River Valley were higher, while the other regions had a low snowfall probability. To study the relationship between altitude and snow cover, the normalized snow elevation correlation index (NSACI) was calculated. The NSACI showed a significant correlation between snow cover and elevation in most regions of Xinjiang and was classified into five grades. Snow cover days did not fluctuate obviously from 2001 to 2015, and a decreasing trend was observed in the four subregions except for the Ili River Valley (nonsignificant decreasing trend). We also observed a correlation between snow cover and temperature and found that the correlations between monthly snow cover and monthly temperature in the four subregions were strongly related to the underlying land type and global warming background, which also suggests that the special topography of Xinjiang greatly influences both snow cover and climate change.

## Introduction

Snow cover is the most important component of the cryosphere, and it significantly influences global and regional radiation balances and atmospheric circulation (Li, Chen & Li, 2019). Snow cover is also a parameter that is sensitive to global environmental changes (Xin et al., 2015). In terms of area, snow cover can reach 4.6 × 10^7^ km^2^ over the Northern Hemisphere during winter, when the snow cover percentage is approximately 18% (Fairman & Anthony, 2014; Liu, Ke & Shao, 2015). In additional, snow cover is also an essential freshwater resource in arid and semi-arid regions (Ye & Grimm, 2013). The relationships and feedback between snow cover and the local climate system could be used to predict global and regional climate change (Kim et al., 2015). The spatiotemporal variability of snow cover significantly affects the heat exchange between the terrain and atmosphere (Zhang et al., 2013). Thus, variations in snow cover can affect regional climate patterns, especially in vulnerable ecological environments (Chen et al., 2012; Schuetz et al., 2001). Changes in snow cover are also closely related to local water resources, and the detection and assessment of temporal and spatial variabilities in local snow cover can provide information for agricultural, ecological, disaster prevention and other related research fields (Seidel et al., 2016).

The Xinjiang Uygur Autonomous Region (Xinjiang) is a typical arid and semi-arid region that lacks significant water resources, and snow is the main water resource in region. Due to the terrain and landform, the snow cover distribution is not balanced among the different regions and seasons in Xinjiang (Zhang, Liu & Han, 2007). In Xinjiang, except for permanent glaciers on the mountains, seasonal snow cover is mainly formed during autumn and winter (from October of one year to April of the following year) (Liang et al., 2008). In addition to agricultural irrigation in spring and summer, during winter months the snow cover also possesses characteristics of heat preservation and thermal insulation and retains soil layer heat, exerting a vital effect on crop wintering (e.g., winter wheat) (Zhang et al., 2013). Hence, snow meltwater is necessary for normal growth in the arid region of Xinjiang.

Related research has shown that snow cover data were mainly obtained from weather stations in various regions (Derksen, Brown & Wang, 2008; Wever et al., 2014). However, in remote regions, weather stations cannot be established due to the terrain, which greatly restricts the acquisition of snow data. Remote sensing is a promising alternative approach to conventional methods of monitoring snow cover and presents high efficiency, low cost, large scale and rapid data acquisition. Therefore, utilizing remote sensing technology for snow cover is a focus of cryosphere sciences, and it has attracted considerable attention in recent years.

In 1999, NASA (the National Aeronautics and Space Administration) launched the Terra satellite, which carries a MODIS (Moderate-resolution Imaging Spectroradiometer) sensor. As primary data, MODIS snow products have been widely used in snow research in various regions around the world (Malmros et al., 2018; Tekeli et al., 2005; Toure et al., 2018) due to high temporal and spatial resolutions. Saavedra et al. (2018) used MODIS data to calculate snow persistence as a fraction of time with snow cover for each year between 2000 and 2016; Dariane, Khoramian & Santi (2017) used MODIS snow cover products, which have been demonstrated to provide a spatially distributed and accurate mapping of snow; Li et al. (2017) analysed the snow cover in Hengduan Mountains based on 500 m resolution MODIS products from 2000 to 2014; Déry et al. (2005) used MODIS data to perform a hydrometeorological numerical simulation on the Kuparuk Basin in Alaska by plotting the snow cover decline curve, and good results were obtained; Bormann, McCabe & Evans (2012) used MOD10A1 products from 2001 to 2010 to generate large-range partitioned snow datasets on a daily scale in an Australian region; Tang et al. (2013) used MOD10A1 products to analyse the temporal and spatial variations of snow cover in the Qinghai-Tibet Plateau from 2001 to 2011; Maskey, Uhlenbrook & Ojha (2011) used the MOD10A2 product and temperature data to study the temporal and spatial variations of snow cover in the Himalayas; and Pu, Xu & Salomonson (2007) applied the MOD10C2 product to analyse the factors influencing snow cover in the Qinghai-Tibet Plateau between 2000 and 2006, such as gradient, slope and curvature.

However, while the studies above have used different MODIS snow products for regional analysis, very few of them address clouds. They simply analysed snow cover percentage and lack the extraction of other snow parameters, such as snow cover days, the rate of snow occurrence and so on. In additional, related study about changes in snow cover caused by other factors has not been conducted. Most importantly, a systematic and comprehensive study on the remote sensing observations of snow in Xinjiang with the MODIS products has not been performed, and the relationship between temperature, elevation and snow cover in arid region requires analysis. Hence, we examined the spatiotemporal variations of snow information in Xinjiang to further explore the relationship between snow cover and climate and elevation factors using 8-day composite products from MODIS (MOD10A2 and MYD10A2).

Xinjiang has approximately 570 rivers, which primarily originate from the three major mountains with an average elevation of 4,000 m (Zhang, Liu & Han, 2007), and the water cycle system in this is an important part of the climate system. Xinjiang is one of the five major snow regions in China, and snow cover is the main process of the water cycle (Li, Chen & Li, 2019). Because the melting of snow at different elevations has a greater relationship with solar radiation, the snow cover at different elevations is also different. We tried to build an index for elevation and snow cover to quantify the relationship between elevations and snow cover. It will provide a convenient tool to reveal the rate of snow cover change in each elevation and be great of significance to the mutual feedback of regional climate change and water cycle in a macroscopic way.

## Materials and Methods

### Study area

**Figure 1:**
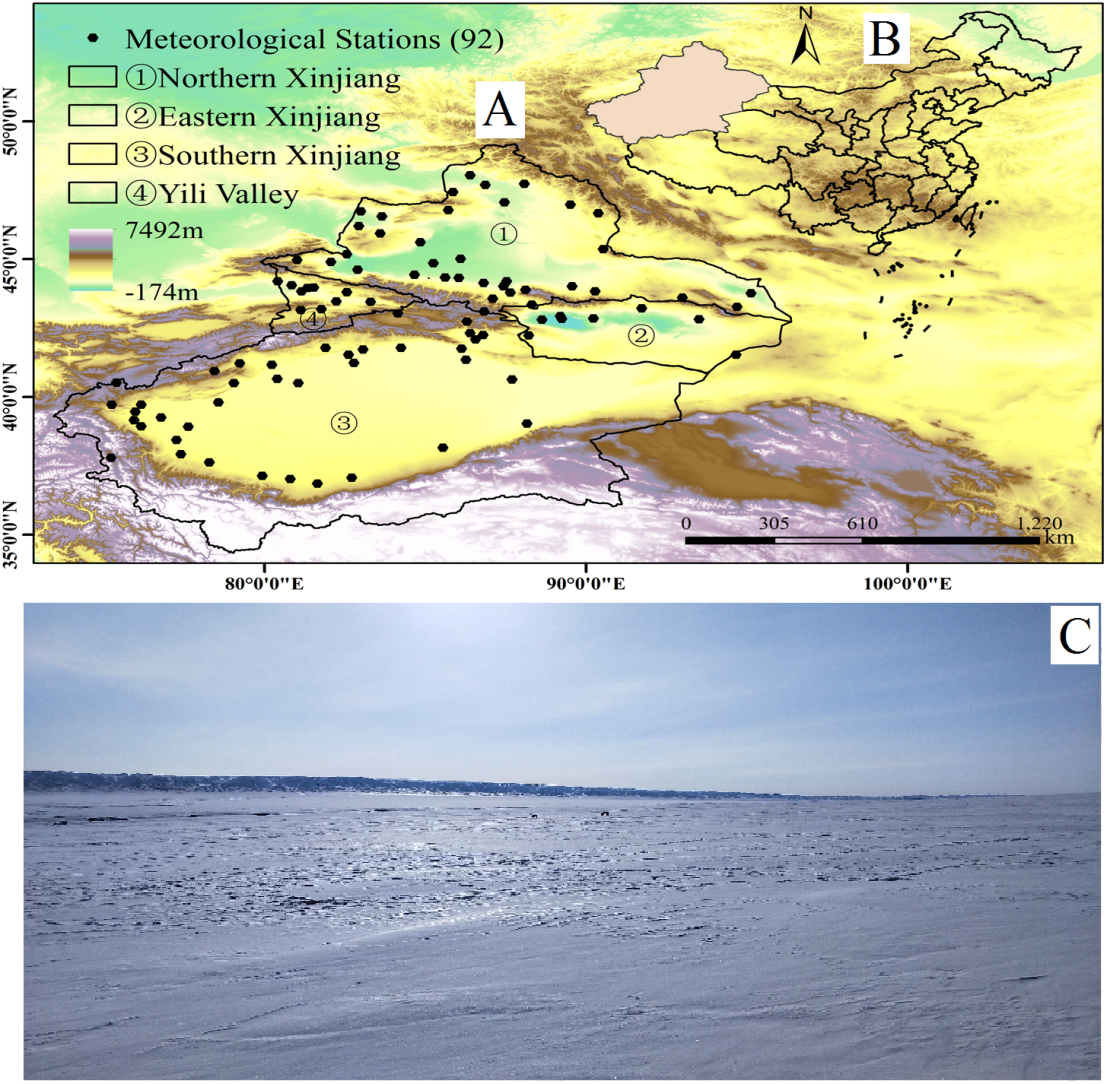
Location of Xinjiang, China, with weather stations and photographs of field sites in Xinjiang. (A) The location of Xinjiang and weather stations, (B) location of China, and (C) photographs of the field sites in Xinjiang (map and photograph credit: Wenqian Chen).

Located in the hinterland of the Eurasian continent, Xinjiang is China’s largest provincial administrative region and an important channel of the ancient Silk Road (Li et al., 2015b) (Fig. 1B). With an east–west length of 1,950 km, north-south width of 1,550 km and area of 1.66 million km^2^, Xinjiang accounts for approximately 1/6 of China’s area. The Altai Mountains lie in the northern part, the Kunlun Mountains are in the southern part, and the Tianshan Mountains span the central area. Xinjiang neighbours eight countries, including Russia, Kazakhstan and Kyrgyzstan. The most significant landform in Xinjiang is characterized by an alternating distribution of mountains (mainly subalpine and alpine mountains) and basins (mainly middle elevation plains), which form a mountain-basin system (Huai et al., 2018). The Tianshan Mountains divide Xinjiang into two parts, with the northern part encompassing the Junggar Basin between the Altai Mountains and Tianshan Mountains and the southern part encompassing the Tarim Basin between the Kunlun Mountains and Altun Mountains. Typically, the area south of the Tianshan Mountains is called Southern Xinjiang, the area north of the Tianshan Mountains is called Northern Xinjiang, and the Hami and Turpan basins are known as Eastern Xinjiang. Xinjiang is located in the westerly belt of the world, and it shows typical temperate continental climatic characteristics. As an inland area surrounded by high mountains and located far from the ocean, the region is not easily reached by ocean moisture. In addition, temperatures vary in different regions; however, the sunshine duration is sufficient (annual sunshine hours of 2,500∼3,500 h). The annual average temperature is 10−13 °C in Southern Xinjiang and less than 10 °C in Northern Xinjiang. The extreme maximum temperature has reached 48.9 °C in Eastern Xinjiang’s Turpan Basin while an extreme minimum temperature of −51.5 °C occurs in Northern Xinjiang’s Keketuohai (Fang et al., 2016). The precipitation in Xinjiang is low overall, with an average annual precipitation of approximately 150 mm and a distinct regional difference. The average precipitation is 20–100 mm in Southern Xinjiang and 100–150 mm in Northern Xinjiang (Xin et al., 2015). Depending on the different climate types, Xinjiang is divided into four regions: Southern Xinjiang, Northern Xinjiang, Eastern Xinjiang and Northern Xinjiang’s Ili River Valley region (Fig. 1A). Figure 1C is a photograph of the snow cover in Xinjiang.

### Data and methods

#### MODIS data

The products provided by MODIS include land data products, atmospheric products and marine products. In addition, MODIS snow cover products are also provided through a series of algorithms (Dorothy & George, 2007). The snow cover data are from the National Snow and Ice Data Center (NSIDC, https://nsidc.org/). MODIS Terra/Aqua 8-day snow cover products consist of MOD10A2 and MYD10A2, and they are projected in an equal-area sinusoidal projection with a spatial resolution of 500 m. According to the difference in spectral reflectance of ice-snow between visible and shortwave infrared bands, snow cover is identified using the normalized differential snow index (NDSI) and threshold discrimination method (Hall et al., 2002). MOD10A2 (MYD10A2) is synthesized by the daily snow product data MOD10A1 (MYD10A1), which represents the maximum snow cover within an 8-day period. In other words, a pixel is marked as snow if snow appears on at least one of the 8 days within that pixel. Different pixel values represent different types of surface features, e.g., 25 for no snow cover; 37 for lake; 50 for cloud; 100 for lake ice and 200 for snow cover (Dorothy & George, 2007).

The MOD10A2 and MYD10A2 data ranged from January 1, 2001, to December 31, 2015. To cover Xinjiang region, data with tile numbers h23V04, h23V05, h24V04, h24V05, h25V04 and h25VV05 were downloaded. Interactive Data Language (IDL) version 8.3 combined with MODIS image processing tool MTCK (MODIS Conversion Toolkit) were used to perform batch processing, including stitching, stacking, re-projection and clipping. For all preprocessed data, the uniform UTM (Universal Transverse Mercator) projection and WGS 84 (World Geodetic System 1984) ellipsoid coordinate system were adopted.

Many studies have validated the accuracy of MODIS snow products. Zhou et al. used hydrological flow and snow cover monitoring data to verify the MOD10A2 product in the Shangnuoli River Basin of the United States, where satisfactory results with a total accuracy of over 83.6% were obtained (Zhou, Xie & Hendrickx, 2005). Pu, Xu & Salomonson (2007) identified snow cover in the Qinghai-Tibet Plateau based on MOD10A2 and obtained a total accuracy of approximately 90%. According to Hall et al., MOD10A2 produced a deviation when the snow depth was <1 cm; however, this finding suggested that MOD10A2 remains applicable to temporal and spatial variation research (Dorothy & George, 2007).

#### Shuttle Radar Topography Mission digital elevation model data

The Shuttle Radar Topography Mission (SRTM) was launched by NASA in 2000 and has obtained altitude data for most parts of the world. The SRTM is an international research effort that obtained digital elevation models on a near-global scale from 56°S to 60°N and was launched to generate the most complete high-resolution digital topographic database of Earth. Because of the high temporal resolution of 1 s (30 m along the equator), the data produce an important optional dataset in the current digital elevation model (DEM). In this study, the 4.1 version of the data was used to ultimately obtain the DEM data within the Xinjiang through stitching, re-projection and clipping with ArcGIS software version 10.1.

#### Meteorological dataset

China Meteorological Assimilation Driving Datasets for the SWAT model (CMADS) covering the period from January 1, 2011, to December 31, 2014, were also obtained. Employing Chinese land data assimilation system (CLDAS) technology, the CMADS datasets provide coverage for all of East Asia and are established by multiple methods, including loop nesting, resampling, pattern estimation and bilinear interpolation, which ensure the applicable accuracy (Wang et al., 2018). In this study, snow depth data from 93 weather stations were utilized to assess the cloud removal precision, and the relationship between climate and snow cover between 2001 and 2014 was analysed based on CMADS meteorological data.

### Methods

#### Snow cover day

Snow cover is used to reflect the spatial distribution of snow within a specific time interval. Snow cover days (SCDs) are used to represent the number of snow cover days per pixel in image. Accordingly, the cumulative SCD value of each pixel during a specific period can be calculated by Eq. (1) (Wang et al., 2014):

(1) \begin{eqnarray*}SCD=8\times \sum _{i=1}^{n} \left( {S}_{i} \right) \end{eqnarray*}

where *S*_*i*_=1 indicates the presence of snow cover, *S*_*i*_=0 indicates the absence of snow cover, and *n* is the time interval. The SCD values from 2001 to 2015 were calculated in the study.

#### Snow cover percentage

The snow cover percentage (SCP) is the ratio of the number of pixels identified as snow to the total number of pixels in a study region, which is expressed as a percentage. According to SCP, we put forward a new definition. In a given period, the number of images remaining after cloud removal fusion is fixed. For each pixel, the percentage of time with snow appearance within a given period is defined as the snow cover appearance percentage (SCAP) (Eq. (2)). If a certain pixel in all MODMYD images shows the appearance of snow, then SCAP = 100%. If there is no snow appearance for a certain pixel in any MODMYD image, then SCAP = 0%. A higher SCAP value indicates a larger percentage of snow appearance for a pixel in a given period, whereas a lower SCAP value indicates a smaller percentage of snow appearance.

(2) \begin{eqnarray*}SCAP= \frac{{P}_{snow}}{P} \times 100\text{%}\end{eqnarray*}

*P*_snow_ denotes a pixel that is identified as snow, and P denotes the number of MODMYD images during a particular period. The SCAP values for the annual seasonal snow cover are subsequently calculated.

In general, regions with higher elevations are covered with thicker snow. However, a complex relationship is observed between snow cover and elevation due to the influence of multiple factors. To analyse this relationship, we assumed that the elevation and SCAP are linearly correlated during the seasonal snow cover period to calculate the approximate relationship between elevation and SCAP, and normalized snow elevation correlation index (NSACI) (Liu, Ke & Shao, 2015) (Eq. (3)) is defined as follows:

(3) \begin{eqnarray*}NSACI=1-abs \left\vert \frac{ \left( SCAP-SCA{P}_{\mathrm{min}} \right) }{ \left( SCA{P}_{\mathrm{max}}-SCA{P}_{\mathrm{min}} \right) } - \frac{ \left( H-{H}_{\mathrm{min}} \right) }{ \left( {H}_{\mathrm{max}}-{H}_{\mathrm{min}} \right) } \right\vert \end{eqnarray*}

In Eq. (3) SCAP represents the period of seasonal snow SCAP (from October of one year to April of the following year) from 2001 to 2015 in Xinjiang, and H denotes the elevation of Xinjiang. The dimensionless values of SCAP and elevation are normalized, and the value range is set at 0–1. Then, the absolute value is taken for the D-value between SCAP and altitude. If NSACI is closer to 1, then both SCAP and altitude may be very high or low at that time. In contrast, if NSACI is closer to 0, then either the SCAP or elevation may be very high or low at that time.

#### Coefficient of variation

The snow cover variation trend in Xinjiang over the years was analysed according to the SCAP in different study area subregions. The variation in amplitude and variability were described by calculating the standard deviation (SD) (Eq. (4)) and its coefficient of variation (CV) (Eq. (5)) in each subregion over the years (Li et al., 2015b). The computational formulas for SD and CV are as follows:

(4) \begin{eqnarray*}& SD=\sqrt{ \frac{\sum _{i=1}^{n} \left( SCD-SC{D}_{mean} \right) }{n-1} }\end{eqnarray*}

(5) \begin{eqnarray*}& CV= \frac{SD}{SC{D}_{mean}} \times 100\text{%}\end{eqnarray*}

#### Trend variation

Linear regression was used to calculate the SCD variation trend from 2001 to 2015, with the SCD of each pixel as the unit (Eq. (6)).

(6) \begin{eqnarray*}slope= \frac{ \left[ n\sum _{i=1}^{n}i\times {x}_{i}- \left( \sum _{i=1}^{n}i \right) \left( \sum _{i=1}^{n}{x}_{i} \right) \right] }{ \left[ n\sum _{i=1}^{n}{i}^{2}-{ \left( \sum _{i=1}^{n}i \right) }^{2} \right] } \end{eqnarray*}

where *n* is the number of monitoring years; *x*_*i*_ is the SCD for the *i* th year; and slope represents the trend variation of SCD over multiple years. The fit line can reflect the variation trend and amplitude of SCD in Xinjiang from 2001 to 2015. A positive slope indicates an increase in SCD, and vice versa.

#### Cloud removal

**Figure 2:**
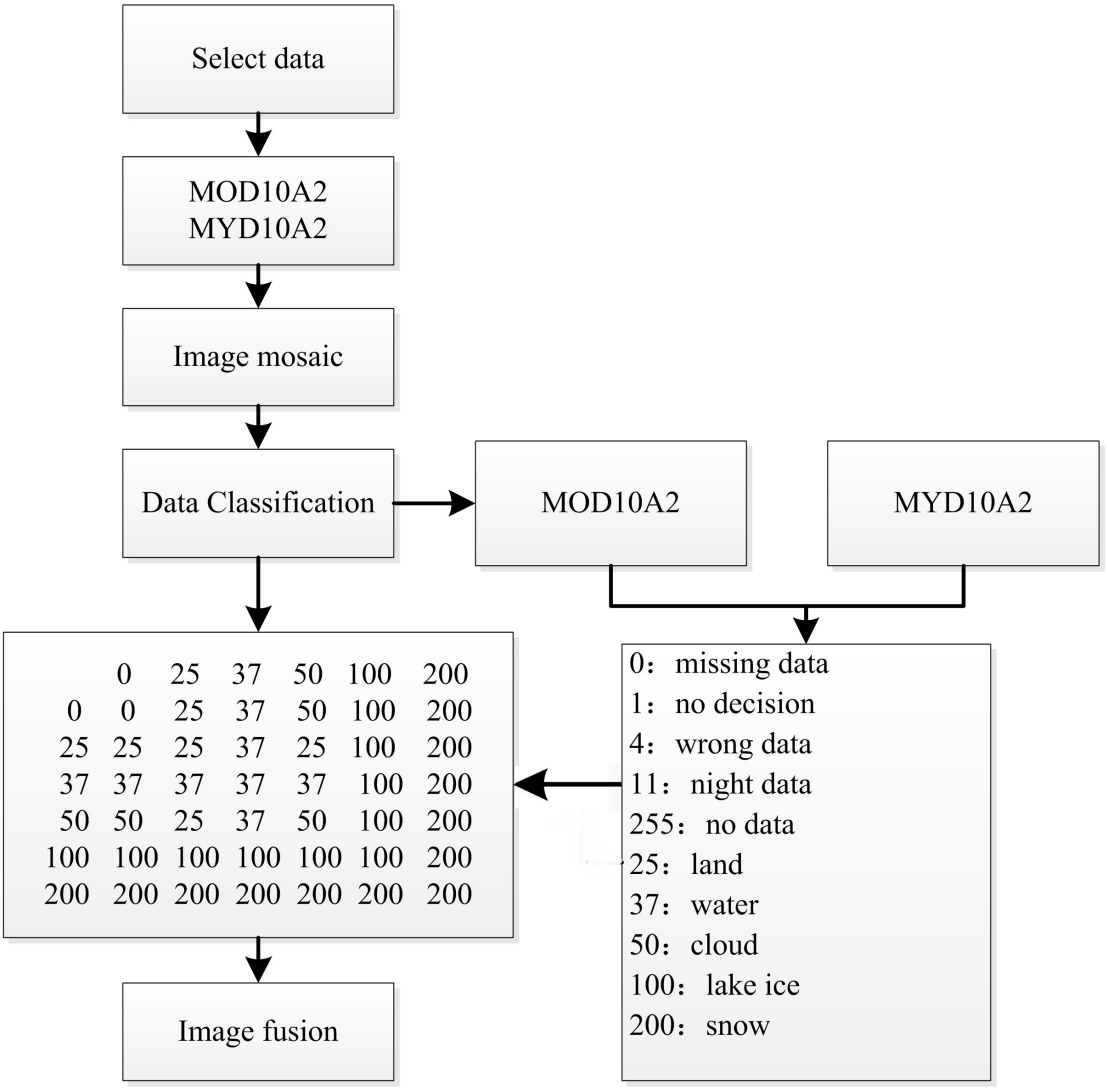
Combination flowchart of removing cloud.

Clouds are the main obstacle for snow monitoring via optical in remote sensing. Although the 8-day snow products could reduce the influence of clouds to some extent, from 2001–2015 41 satellite images of MOD10A2 in the study area have cloud coverage exceeding 10%, accounting for 6% of all data. Additionally, the majority of the 41 remote sensing images obtained during the winter months may influence the accurate extraction of snow cover information. In this study, clouds were removed by fusing MOD10A2 with MYD10A2 (Xie, Wang & Liang, 2009; Zhang et al., 2017). The main principles of removing clouds are as follows: with MOD10A2 and MYD10A2, all pixel values are defined as zero except for clouds (50), snow (200), lake ice (100), land (25) and water (37). If a pixel is identified as a cloud in one of two data sets (MOD10A2 or MYD10A2) but identified as snow, land or water in another data at the same time, then the pixel will be recognized as the other feature rather than as a cloud. So, two data complementing each other at one time can remove clouds. The combined algorithm can be expressed as follows and is shown in Fig. 2.

 Through the fusion and matching of two image sets (with clouds) for the same period, the 41 images with over 10% cloud coverage were eventually reduced to 12 images, thereby reducing the influence of clouds on the extraction of snow cover information in Xinjiang.

After cloud removal by fusing MOD10A2 and MYD102, the recognition rate of snow cover was effectively improved. The precision of the fused declouded snow cover data was examined based on the daily snow depth data from 93 ground weather stations in Xinjiang. The snow data observed by weather station are in cm, snow depth <0.5 cm was set as no snow cover, and snow depth ≥0.5 cm was set as snow cover (Zhang et al., 2008). The total precision *P* (Eq. (7)), omission classification rate *D* (Eq. (8)) and misclassification rate *V* (Eq. (9)) are considered to be the basis for assessing the precision:

(7) \begin{eqnarray*}& P= \frac{A+B}{T} \end{eqnarray*}

(8) \begin{eqnarray*}& D= \frac{X}{T} \end{eqnarray*}

(9) \begin{eqnarray*}& V= \frac{Y}{T} \end{eqnarray*}

where *A* is the number of samples with snow presence for both images and ground stations; *B* is the number of samples without snow for both images and ground stations; *T* is the total number of samples; *X* is the number of samples without snow for the image data but with snow for ground stations; and *Y* is the number of samples with snow presence for the image data but without snow for the ground stations. For the fused images, the overall precision of snow cover information reached 94.2% (Table 1).

**Table 1:** Data accuracy analysis of MODIS.

Meteorological data	MODIS data			Converged data		
	Snow	No snow	Total	Snow	No snow	Total
Snow	*A* = 2921	*X* = 855	3,776	*A* = 2956	*X* = 802	3,758
No snow	*Y* = 924	*B* = 17214	18,138	*Y* = 478	*B* = 17678	18,156
Total	3,434	18,480	21,914	3,434	18,480	21,914
Evaluation accuracy	*P* = 92.0%	*D* = 3.9%	*V* = 4.2%	*P* = 94.2%	*D* = 3.7%	*V* = 2.1%

## Results

### Monthly SCP variations

**Figure 3:**
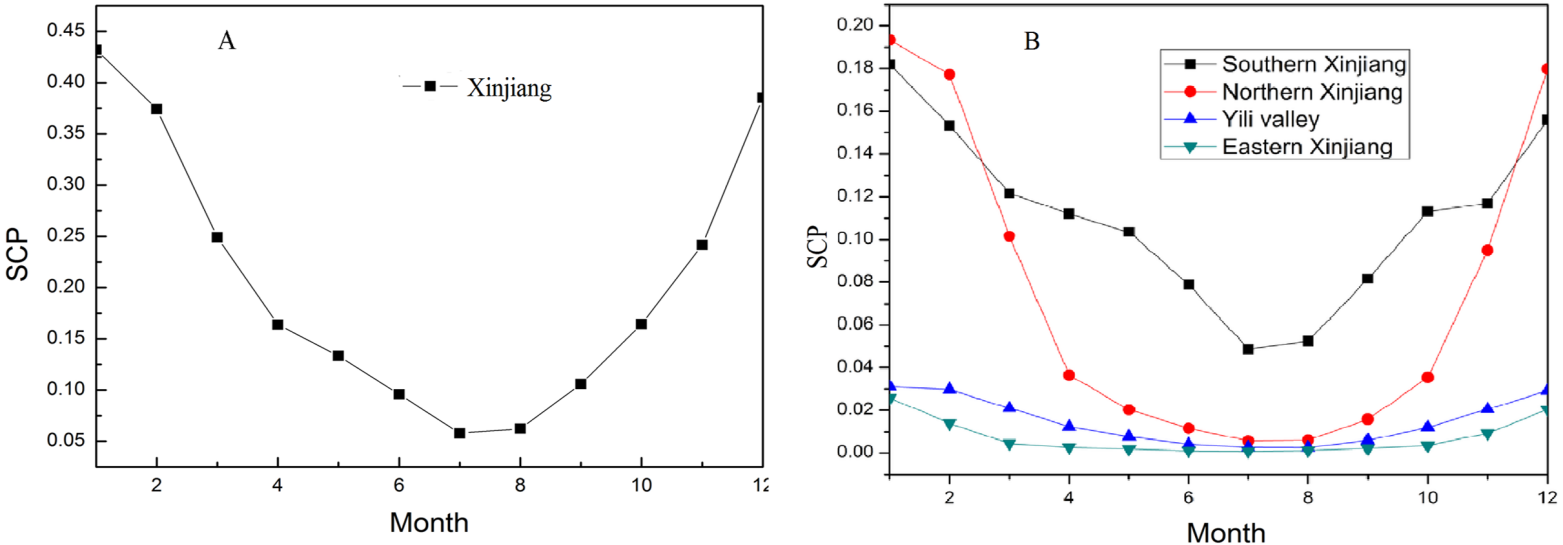
Annual cycles variations in SCP (%) for Xinjiang from 2001 to 2015. (A) All of Xinjiang. (B) The four climate subregions.

Xinjiang was divided into four subregions for analysis, and the SCP of each region was calculated based on the total area of Xinjiang; thus, the total SCP of Xinjiang is the sum of four subregions. As shown in Fig. 3A, a larger SCP appears in December and January while a lower SCP appears in July and August of each year in Xinjiang, and during the seasonal snow period (from October of one year to April of the following year), the average SCP over the 15-year period (during 2001–2015) can reach 37.3%. Among them, snow accumulates each year from mid-October to the following February, and during this period SCP continues to grow, peaking (43.2%) in January, remaining a gradual decline to 37.4% in February smoothly, and beginning to decline rapidly (5.8%) from March to April.

Figure 3B illustrates variations in SCP of the four subregions, overall, it presents similar trends. Between February and April, snow ablation presents a rapid decline in both subregions as well as difference in region, and the difference is mainly caused by elevation. For example, the Ili River Valley is situated north of the Tianshan Mountains, it exhibits a certain similarity to Northern Xinjiang, however, it has higher average elevation and an earlier snow accumulation than other part of Northern Xinjiang. Despite the SCP (18.1%) in Southern Xinjiang being high in overall (this region has largest proportion of area in Xinjiang and Kunlun Mountain and Pamir Plateau are located in the region), the intra-annual SCP variation differs significantly from the other three subregions due to influences of extremely low average precipitation, and extremely high average temperature because of Taklimakan Desert. We also found that the SCP decreases quickly in March for Eastern Xinjiang, where the snowmelt process occurs one month earlier than in Northern Xinjiang and the Ili River Valley, and after March the snow cover remains unchanged.

### Interannual variations in SCP

**Figure 4:**
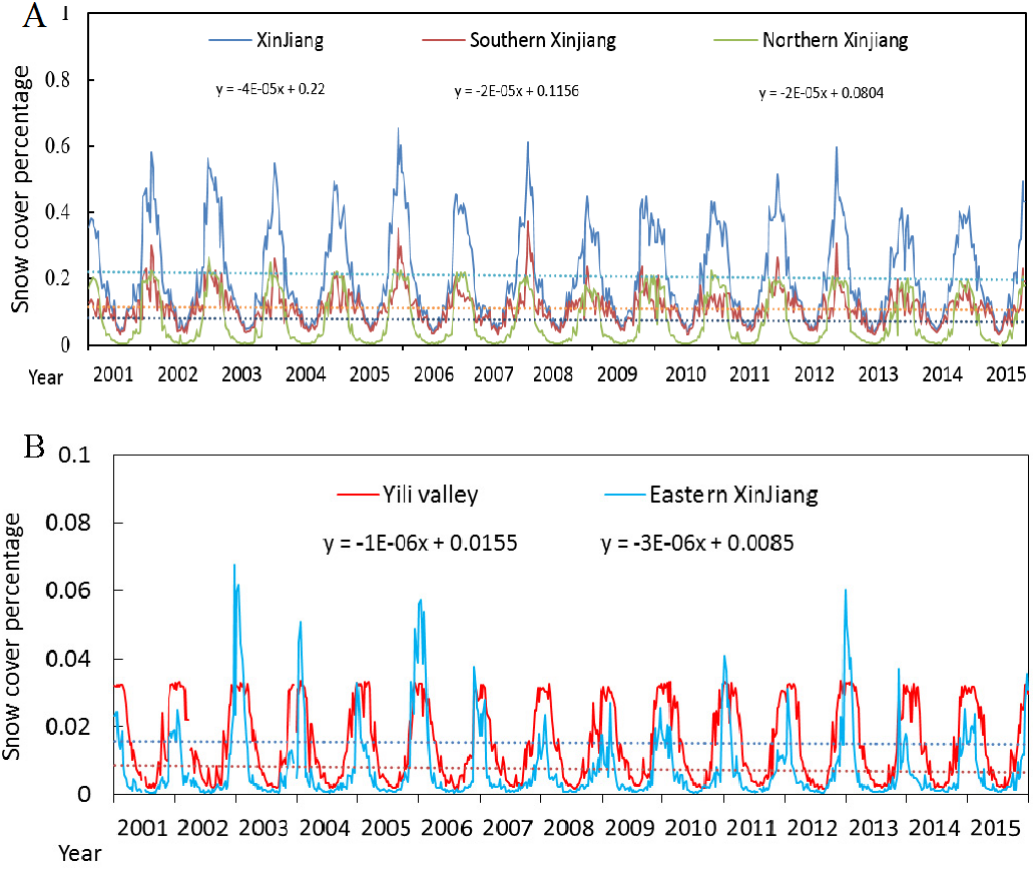
Plot of interannual changes in the SCP of Xinjiang. (A) Northern Xinjiang, Southern Xinjiang and the whole Xinjiang. (B) Eastern Xinjiang and the Ili River Valley.

To quantitatively analyse the interannual and seasonal variations in SCP in Xinjiang, SCP was superimposed with subregional vector files (the vector files are the boundary of each region) to derive the time series of SCP variations in Xinjiang from 2001 to 2015 (Fig. 4). Before the performing trend analysis shown in Fig. 4, the data were subjected to significance verification of the regression trends. The test found that in five regions *p* > 0.05, and the R^2^ values ranged from 0.0013 to 0.0057, indicating that all of the SCP changes in the five regions from 2001 to 2015 were not significant, and the standard error was between 9 and 17.6. The SCP variation curve of the whole Xinjiang region shows that periodic change is very obvious, and periods of snow accumulation and snow melting occur every year (Fig. 4A). We found that SCP was above 40% for all years, peaking in 2006 (65.3%). For Xinjiang, the SCP variations did not fluctuate obviously and presented a slow decreasing trend. However, for the four subregions, the highest SCP was 37.1% in Southern Xinjiang, followed by 22.6% in Northern Xinjiang and 6.7% in Eastern Xinjiang. Because of the humid climate of the valley and the permanent snow in region, the Ili River Valley exhibited mildest variation among subregions, and a maximum SCP of approximately 3.3% every year (Fig. 4B). We found SCP clearly fluctuated in Eastern Xinjiang, which is mainly attributed to the snowfall instability caused by extreme weather within the region, such as extreme temperatures in Hami and Turpan and so on. In summary, the interannual SCP variations differed by subregion due to the regional climate and the type of underlying surface, however, the periodic variations in SCP of the five regions were all obviously.

**Figure 5:**
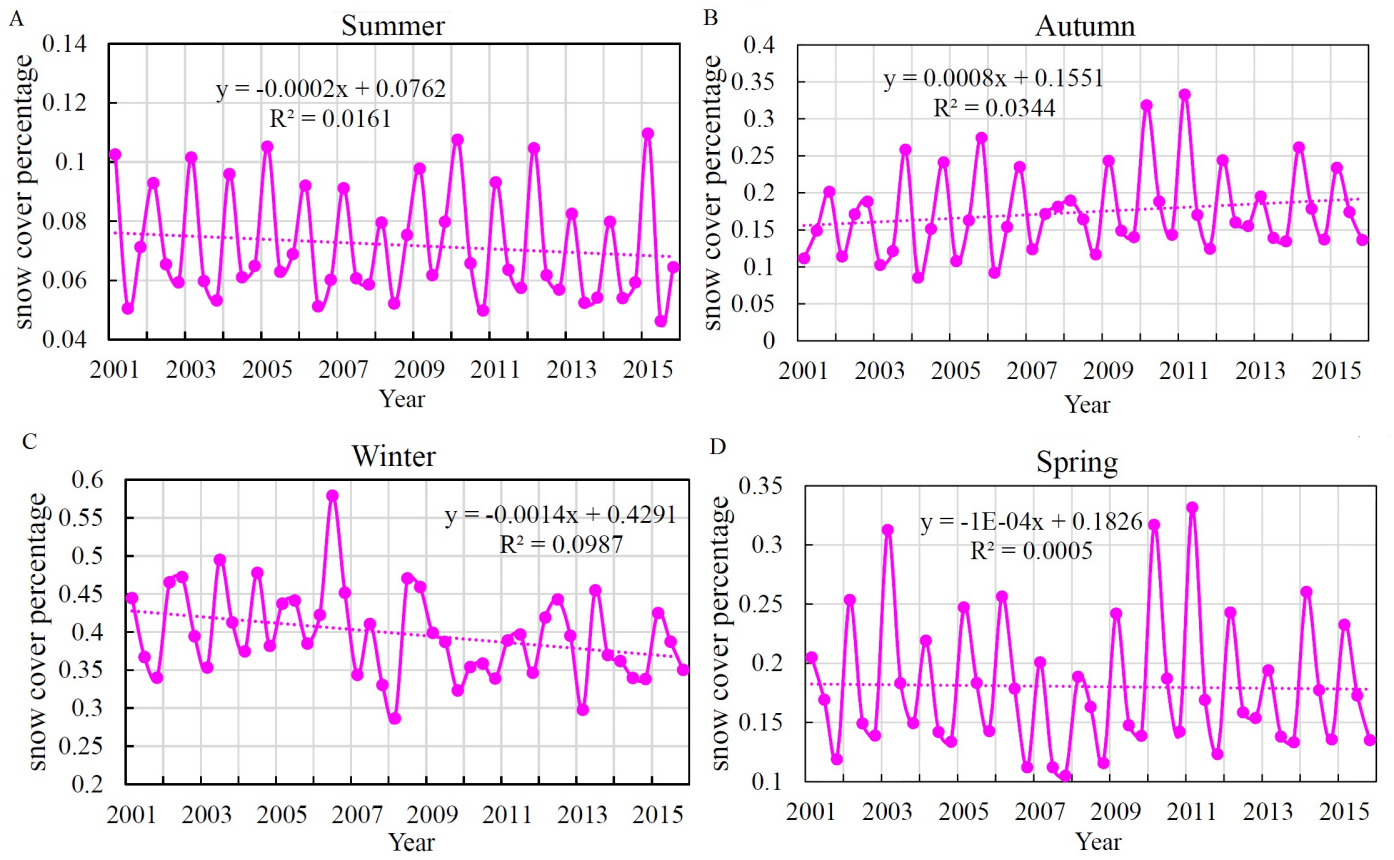
Interannual change in SCP of Xinjiang during different seasons from 2001 to 2015. (A) Summer, (B) autumn, (C) winter, (D) spring.

In this study, each year was divided into four seasons: March to May is spring; June to August is summer; September to November is autumn; and December to February is winter (Guerrero-Rascado et al., 2013). Figure 5 illustrates the SCP variations in spring, summer, autumn and winter from 2001 to 2015. As in Fig. 4, the data were subjected to a significance analysis of regression trends, and de-seasonal cycle was removed. The test found that in three seasons (except for winter) *p* > 0.05, *p* < 0.05 for winter, and the R^2^ range was between 0.003 and 0.12. These trends show that the change in SCP from 2001 to 2015 was more significant in winter, whereas it was not significant in other seasons, and the standard error was between 5.8 and 12.26. The SCP shows a decreasing trend during winter, spring and summer, and the decline is especially evident in winter (Figs. 5A, 5C and 5D), which is likely related to the general context of global warming. The major vapour sources influencing Xinjiang’s atmospheric precipitation are the mid-latitude westerly circulation and the dry cold Arctic airflow (Chen & Dai, 2009). Global warming has resulted in decreased water vapour in southern and central north-western Xinjiang in winter, decreased atmospheric water vapour from northern Xinjiang to western Qinghai and eastern Yellow River in spring and summer, and slightly increased water vapour in central and northern Xinjiang in autumn, which are consistent with the results of this study (Zhao et al., 2017) (Fig. 5B). From the above analysis, it can be concluded that the intra-annual-annual variation of SCP in each region showed both regional integrity and uniqueness. Due to the combined effects of climate and terrain factors, regional differences exist.

### Monthly SCAP variations

**Figure 6:**
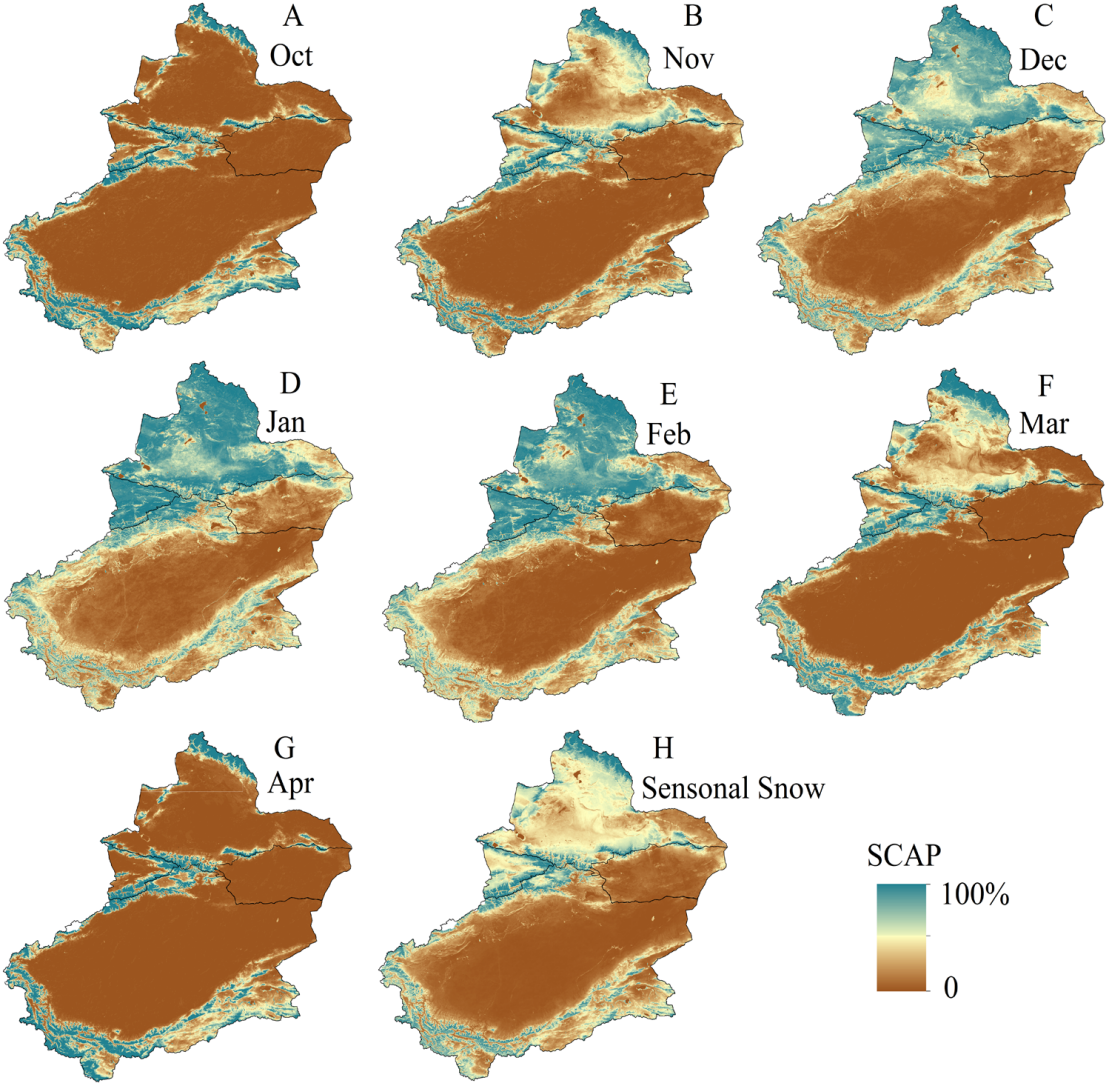
Spatial distribution of SCAP of Xinjiang during period of seasonal snow (from October of one year to April of the following year). (A) October. (B) November. (C) December. (D) January. (E) February. (F) March. (G) April. (H) Seasonal snow (map credit: Wenqian Chen).

The areas with the highest SCAP in Xinjiang are mainly distributed in three mountainous regions (Fig. 6). Xinjiang is demarcated by the Tianshan Mountains, and the places north of the Tianshan Mountains exhibit higher SCAP than those south of the mountains. From October to the following April, the intra-annual SCAP first increases and then decreases (Figs. 6A, 6B, 6C, 6D, 6E, 6F and 6G). In January the SCAP reaches a maximum, whereas in October and April the SCAP is at a minimum.

The SCAP values were generally higher in all of Northern Xinjiang and the Ili River Valley than in the other two subregions, especially between January and February, with over 90% of the locations in Northern Xinjiang and the Ili River Valley presenting SCAPs between 50% and 100% (Fig. 6H). The highest SCAP values in Southern Xinjiang were distributed mainly in the Kunlun Mountains while the lowest SCAP values ranging from 0–30%were found in the Taklimakan Desert region (China’s largest desert) and its margin. The Kunlun Mountain system has a complex terrain, and from west to east it includes the Pamirs of China, the Karakoram between Xinjiang and Kashmir and the Kunlun Mountains between Xinjiang and Tibet. The north branch includes the Altun Mountains, which stretch north-eastward to join the Qilian Mountains. Despite the high average elevation of the Kunlun Mountains, the mountains are adjacent to multiple parallel valleys. The SCAP values between the parallel valleys are low at only 20% in some areas. For Eastern Xinjiang, the distribution of SCAP is similar to that of Southern Xinjiang (low and all within the desert area) except in the southern Tianshan piedmont, where the SCAP is high.

### Interannual variations in SCAP

**Figure 7:**
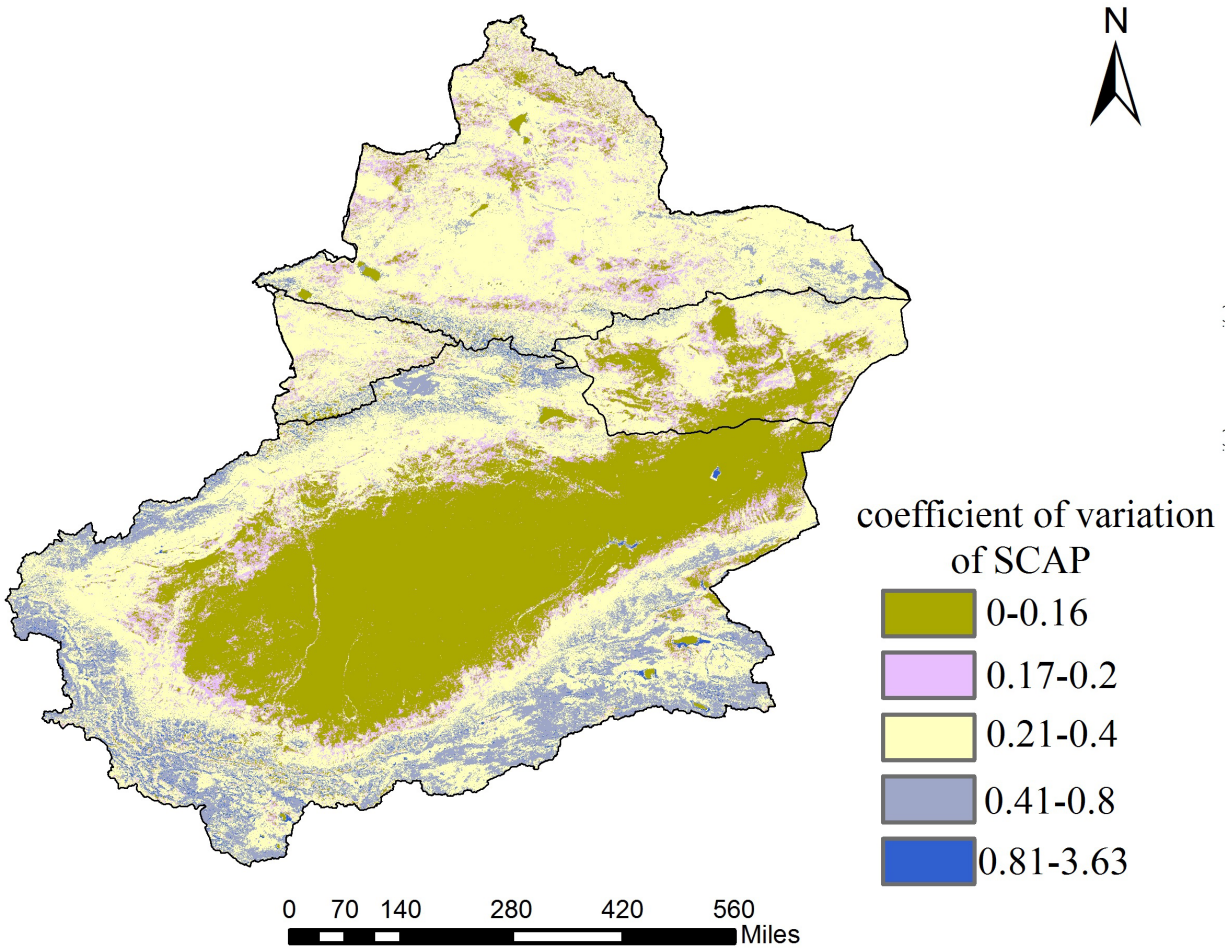
Interannual variations in SCAP of Xinjiang. Map credit: Wenqian Chen.

From 2001 to 2015, the SCAP variability in southeast-southwest-central Xinjiang is greater overall than in the northern part (Fig. 7). The coefficients of variation for Southern Xinjiang desert areas and their margins and for the desert areas in Northern Xinjiang between 0% and 0.2% have little fluctuation. The Southern Xinjiang oasis areas (distributed along the Kunlun-Altun Mountains) and the Northern Xinjiang Junggar Basin exhibit moderate variability, with coefficients of variation ranging from 0.2% to 0.4%. The variability in the middle section of the Tianshan Mountains in Northern Xinjiang and in the Kunlun-Altun Mountains in Southern Xinjiang is large, with coefficients of variation ranging between 0.41% and 0.8%. In summary, the higher SCAP variability in the Xinjiang region is mainly concentrated in the Kunlun Mountains and Tianshan Mountains, which is greatly related to the accelerated deglaciation of Xinjiang’s glaciers over the past 10 years and the accelerated climate warming caused by the carbon release from thawing permafrost (Wang, Zhang & Ma, 2006).

**Figure 8:**
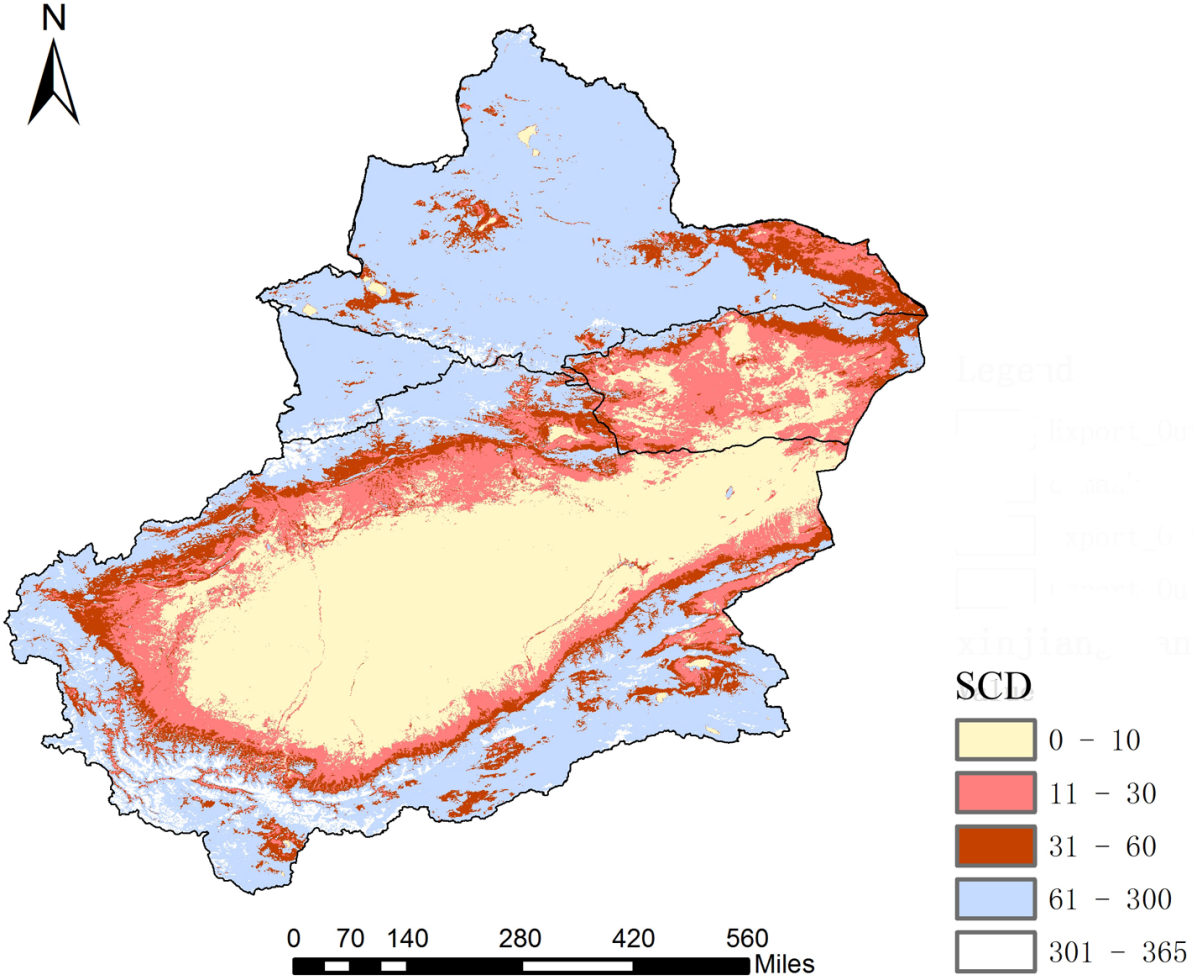
Average SCD from 2001 to 2015 of Xinjiang. Map credit: Wenqian Chen.

The average SCD values of the Xinjiang region between 2001 and 2015 are shown in Fig. 8. The high SCD values are mainly distributed in the three major mountains of Xinjiang while the low values are primarily distributed in the desert areas. Unstable snow (SCD<30 d) is mainly distributed in the Southern and Eastern Xinjiang deserts and their marginal areas, especially in the Eastern Xinjiang region, which is almost completely covered by unstable snow. The stable snow (SCD>30 d) is mainly distributed in Northern Xinjiang, the Ili River Valley area and the Kunlun Mountains in Southern Xinjiang. Northern Xinjiang is a stable snow zone except for Yiwu County, Balikun County, the Ebinur Lake area and the western part of the Junggar Basin. The SCD in the Southern Xinjiang region shows a stratified distribution due to the existence of transitional areas between the oasis exteriors and deserts, which are mainly located between the northern Kunlun piedmont periphery-northern Altun piedmont and the Taklamakan Desert as well as from the Aksu Oasis to the Taklamakan Desert and from the Turpan region to the Taklimakan Desert. The overall trend is a sequential increase in SCD from deserts to oases and then to mountains.

**Figure 9:**
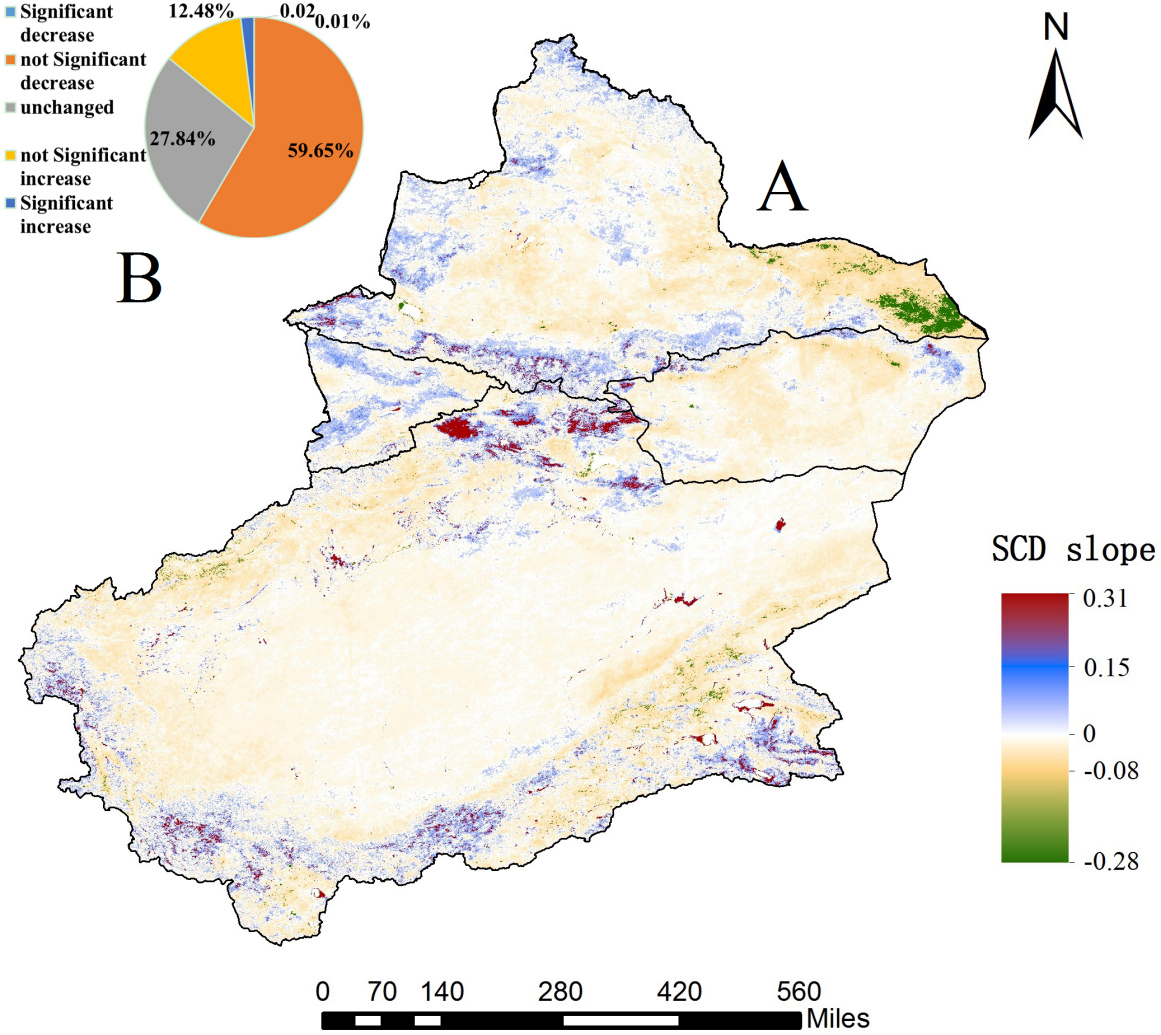
Variations in SCD trend from 2001 to 2015 of Xinjiang (at a significance level of *p* < 0.05). (A) Variations in trend of SCD of Xinjiang. (B) Significance level of SCD trend of Xinjiang (map credit: Wenqian Chen).

The average SCD values were counted annually, and the trends in SCD in Xinjiang were calculated according to trend formula from 2001 to 2015 (Fig. 9A). A slope of >0 indicates an increase in SCD; <0 indicates a decrease in SCD; and =0 indicates no change in SCD. The results reveal that 59.65% of the SCD pixel values in Xinjiang present a non-significant decreasing trend from 2001 to 2015, and only 0.01% of the pixel values show a significant decrease (*p* < 0.05) (Fig. 9B). Change rates between −0.08 and −0.28 are mainly located in Yiwu County, Balikun County and the northern foot of the Altun Mountains. Correspondingly, 12.48% of the SCD pixels present a non-significant increase, and only 0.02% of pixels show a significant increase (*p* < 0.05). Change rates between 0.15 and 0.31 are mainly distributed in the Kunlun and Karakoram Mountains at the Xinjiang-Tibet border and a partial region of the middle Tianshan section. In general, 72.13% of pixels exhibited a weak variation trend (including reduction and increase) while 27.84% of pixels remained stable over 15 years.

**Figure 10:**
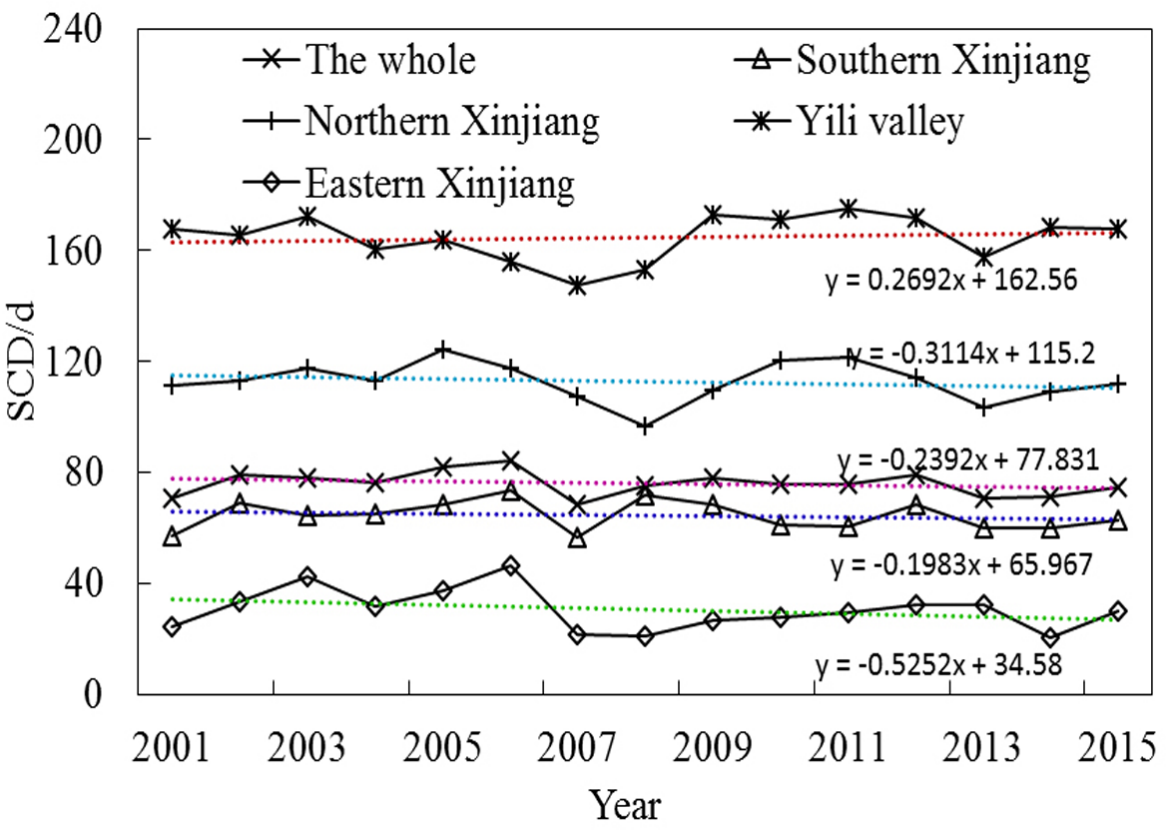
Interannual variation in average SCD for four subregions and Xinjiang.

The statistics of SCD for the analysis trend from 2001 to 2015 were verified before analysis (Fig. 10). R^2^ between 0.02 and 0.09 showed a non-significantly decreasing trend in five regions, and various subregions indicated obvious spatial heterogeneity in the SCD distribution among subregions. The SCD of four subregions except for the Ili River Valley also showed a non-significant decreasing trend. The increase in SCD of the Ili River Valley is related to winter precipitation in the western Tianshan section in recent years (Li, Chen & Li, 2019). Among the four subregions, the Ili River Valley exhibited the highest average SCD, 164 days. The value was 112 days for Northern Xinjiang, 64 days for Southern Xinjiang, 30 days for Eastern Xinjiang and 75 days for the entire Xinjiang region. The SCD fluctuated most remarkably in Eastern Xinjiang and least remarkably in Southern Xinjiang. In the context of persistent climate warming, Xinjiang’s glaciers will continue to ablate. According to the HadCM2 and HadCM3 simulations of global snowfall, the snowfall days and snow cover area in Xinjiang are both decreasing (Chen et al., 2017; Ming et al., 2015). It can be concluded that the annual-interannual variation of SCD is similar to SCP. High mountains are affected by the dynamics and thermal effects of plateaus, and the SCD of mountains is characterized by a vertical zonal gradient. In the different periods of water and heat in plains and basins, the distribution of SCD was latitudinally/non-latitudinally staggered.

### Relationships between SCAP and elevation

**Figure 11:**
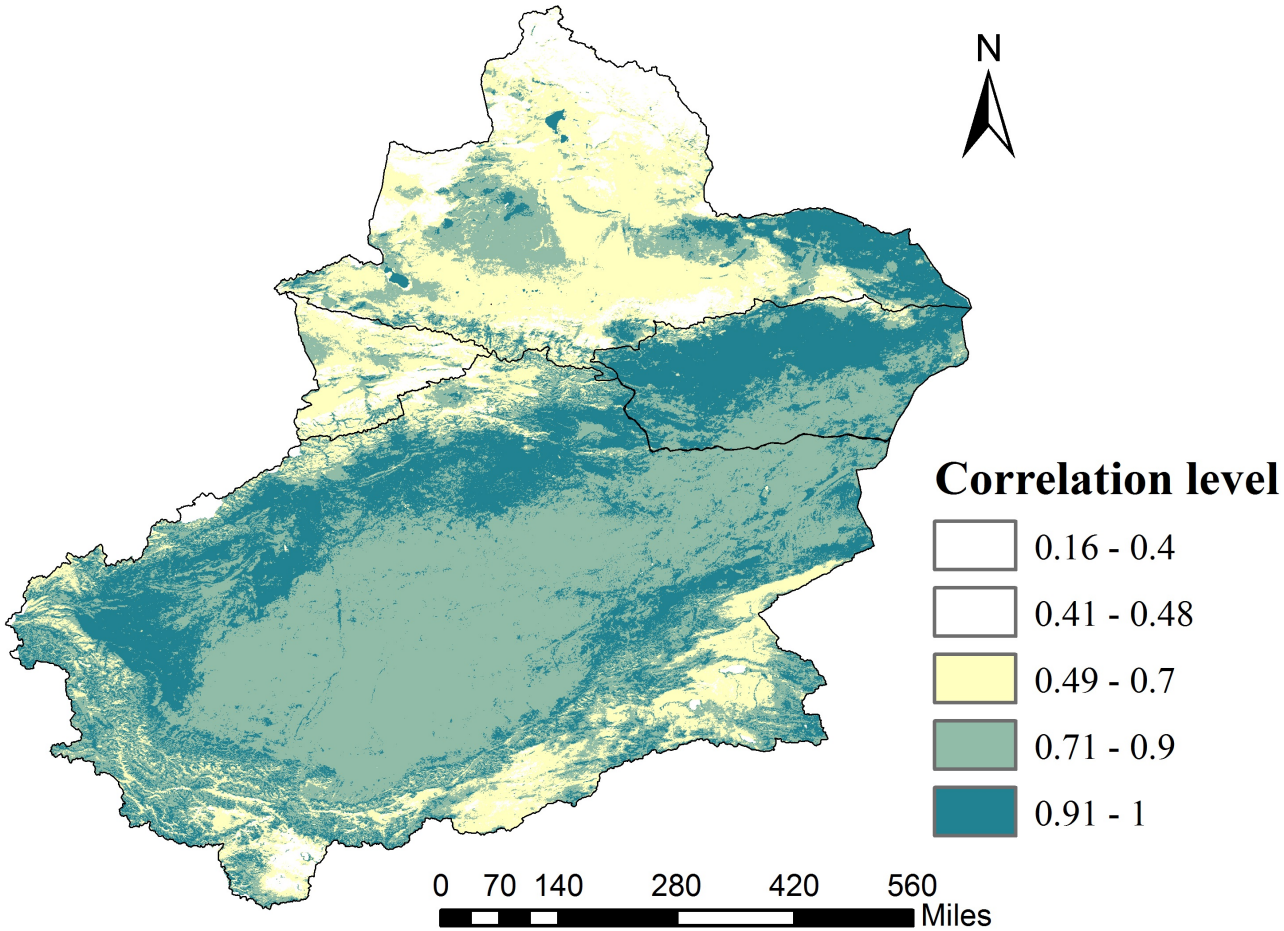
The NSACI in Xinjiang from 2001 to 2015. Map credit: Wenqian Chen.

The high NSACI index suggests that elevation has a great impact on snow cover. In contrast, for areas with low NSACI indices, snow distribution is influenced by other factors, and elevation is not a decisive factor. The NSACI index of Xinjiang was calculated and classified into five grades (Fig. 11): very low correlation (0.16–0.4); low correlation (0.41–0.48); moderate correlation (0.49–0.7); high correlation (0.71–0.9) and a very high correlation (>0.9). The percentages of area occupied by the very low, low, moderate, high and very high correlations are 0.86, 16.84, 11.67, 36.22 and 34.41%, respectively. In the study area, the insignificant area is marked as white (*p* > 0.05), but some areas were significantly correlated (*p* < 0.05); however, the main areas were not significantly correlated with high-altitude glacial regions. Thus, in most parts of Xinjiang, the SCAP is highly correlated with elevation. Regions with an NSACI above 0.8 account for approximately 70% of the total area while regions with an NSACI over 0.6 account for approximately 80%. Low correlations and very low correlations are mainly concentrated in Northern Xinjiang and in the Karakoram and Altun Mountains of Southern Xinjiang. High correlations and very high correlations are primarily concentrated in the Eastern Xinjiang and Southern Xinjiang deserts, oases and some parts of the Karakoram and Altun Mountains. In addition, the low NSACI observed in some areas are determined by factors such as terrain rather than elevation. For example, Qilian Mountain in the Kunlun Mountains system has an elevation exceeding 3,500 m despite a low SCAP. So, we can find that the trend of SCAP at different altitudes is very likely caused by interaction of the mountain effect and atmospheric circulation except for elevation.

### Relationships between the SCP and temperature

The monthly average SCP shows a negative Pearson correlation with temperature from 2001 to 2015. We calculated correlations between monthly snow cover and monthly temperature after removing the seasonal cycle. Table 2 shows the correlation coefficients between the SCP and air temperature in different regions. A significant negative correlation was found between the SCP and air temperature in the whole area of Xinjiang for March, April, May, November and December (*p* < 0.01 or *p* < 0.05). Compared with the other three regions, the months exhibiting high SCP correlations with temperature are fewer in number in the Eastern Xinjiang area and are mainly concentrated in January, April and December. In the Southern Xinjiang and Ili areas, the months showing high SCP correlations with temperature are mainly concentrated in winter and spring, especially from February to May. In the Northern Xinjiang region, the months presenting high SCP correlations with temperature are primarily concentrated in spring, summer and autumn, which also leads to spring and summer floods in the region. In January, February and December, the correlation between temperature and SCP is very low and related to the widespread seasonal snow in Northern Xinjiang. From mid-October to December, the Northern Xinjiang region is part of a snowfall process without snowmelt. At this time, snow already covers most areas; therefore, further temperature reduction will not cause much change in the snow cover, thereby resulting in a very low correlation between temperature and the SCP for the two months. The very low correlation in January in the Southern Xinjiang region is linked to temperature changes in addition to the above reasons. The temperature is fairly high in Southern Xinjiang. In addition to the permanent snow of the Kunlun Mountains, the plains and desert areas receive little snow, and snowmelt also occurs earlier (beginning in February) than in other regions. In summary, the correlation coefficient between air temperature and SCP shows that the difference in the occurrence of snow accumulation and snowmelt processes in various areas caused by different underlying surfaces results in correlation discrepancies between temperature and month.

**Table 2:** Pearson correlation coefficients for the monthly average SCP and temperature in different sub-regions from 2001 to 2015.

Month	Southern Xinjiang	Northern Xinjiang	Eastern Xinjiang	Yili Valley	Whole area
Jan	−0.421	−0.243	−0.604^*^	0.031	−0.356
Feb	−0.674^**^	−0.115	−0.070	−0.493	−0.207
Mar	−0.651^*^	−0.912^**^	−0.410	−0.662^**^	−0.835^**^
Apr	−0.503	−0.836^**^	−0.734^**^	−0.643^*^	−0.638^*^
May	−0.755^**^	−0.312	−0.449	−0.514	−0.567^*^
Jun	−0.252	−0.782^**^	−0.342	−0.416	−0.119
Jul	0.062	−0.468	−0.422	−0.436	0.051
Aug	−0.328	−0.546^*^	−0.431	−0.261	−0.312
Sep	−0.320	0.012	−0.419	−0.390	−0.197
Oct	0.030	−0.163	−0.101	−0.609^*^	−0.045
Nov	−0.496	−0.857^**^	−0.154	−0.847^**^	−0.737^**^
Dec	−0.583^*^	−0.357	−0.635^*^	−0.644^*^	−0.790^**^

**Notes.**

* Significantly correlated at the 0.05 level.

** Significantly correlated at the 0.01 level.

The correlations between the SCP and temperature in various regions for all years show significant negative correlations (all *p* < 0.01) (Table 3). Xinjiang is an important part of the arid region of Central Asia. The clearly continental climate presents four distinct seasons. In winter, the temperature is cold, and the seasonal snow cover is high. In summer, the temperature is high, seasonal snow is completely melted, and only permanent snow in the high-altitude area remains. The period from mid-October to mid-December represents the first stage of the snow accumulation period; mid-December to mid-February of the next year represents the second stage of the snow-stabilization period; and late February to mid-April of the next year represents the third stage of the snowmelt period. Therefore, changes in the SCP are dominated by temperature. In addition, the correlation coefficients are rather high, indicating that considerably stable correlations occur between snow cover and temperature in various regions of Xinjiang. Due to the lack of meteorological data for 2015, only the period from 2001 to 2014 is shown in Table 3.

**Table 3:** Intra-annual Pearson correlation coefficients for SCP and temperature for different subregions of Xinjiang from 2001 to 2015.

Year	Southern Xinjiang	Northern Xinjiang	Eastern Xinjiang	Yili Valley	Whole region
2001	−0.829^**^	−0.965^**^	−0.902^**^	−0.978^**^	−0.963^**^
2002	−0.927^**^	−0.931^**^	−0.812^**^	−0.985^**^	−0.957^**^
2003	−0.783^**^	−0.936^**^	−0.749^**^	−0.979^**^	−0.948^**^
2004	−0.916^**^	−0.957^**^	−0.806^**^	−0.984^**^	−0.962^**^
2005	−0.874^**^	−0.976^**^	−0.852^**^	−0.982^**^	−0.985^**^
2006	−0.938^**^	−0.955^**^	−0.874^**^	−0.968^**^	−0.971^**^
2007	−0.866^**^	−0.937^**^	−0.784^**^	−0.983^**^	−0.956^**^
2008	−0.871^**^	−0.967^**^	−0.922^**^	−0.982^**^	−0.957^**^
2009	−0.903^**^	−0.977^**^	−0.915^**^	−0.98^**^	−0.98^**^
2010	−0.832^**^	−0.973^**^	−0.908^**^	−0.975^**^	−0.976^**^
2011	−0.812^**^	−0.971^**^	−0.865^**^	−0.987^**^	−0.974^**^
2012	−0.838^**^	−0.978^**^	−0.802^**^	−0.993^**^	−0.979^**^
2013	−0.947^**^	−0.974^**^	−0.845^**^	−0.985^**^	−0.971^**^
2014	−0.856^**^	−0.979^**^	−0.809^**^	−0.993^**^	−0.99^**^
2015	–	–	–	–	–

**Notes.**

** Significantly correlated at the 0.01 level.

**Figure 12:**
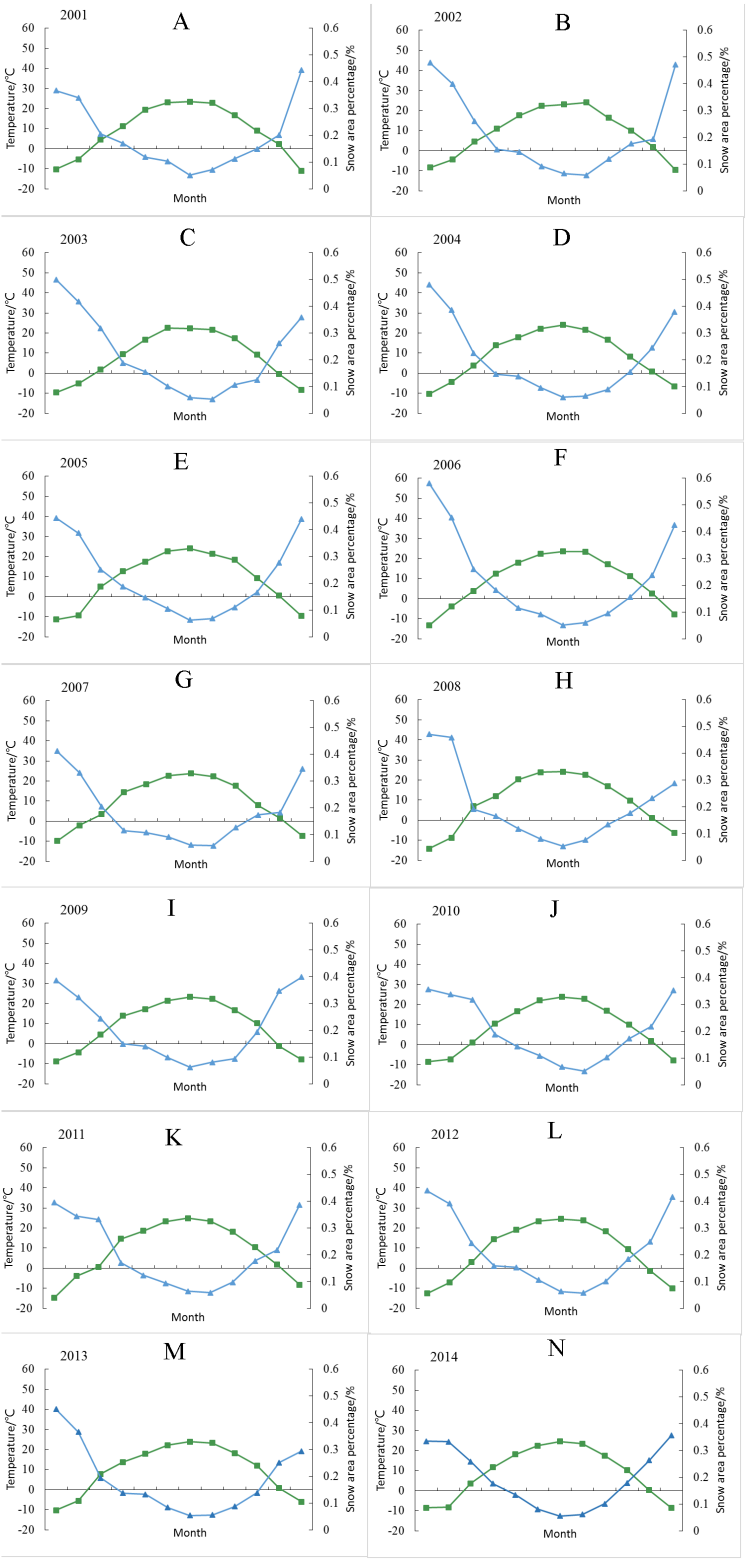
Annual variation between the monthly SCP (%) and temperature of Xinjiang from 2001 to 2014 (all satisfy the significance test at *p* < 0.01). (A) 2001. (B) 2002. (C) 2003. (D) 2004. (E) 2005. (F) 2006. (G) 2007. (H) 2008. (I) 2009. (J) 2010. (K) 2011. (L) 2012. (M) 2013. (N) 2014.

In Figs. 12A–12N, the snow cover decreases with increasing temperature from January to December, and when the temperature decreases, the snow melt process also slows. Beginning in mid-October, the temperature begins to drop to zero, and the SCP begins to increase. Due to the influence of the Mongolian-Siberian High, cold air enters the Xinjiang region in November, which leads to an average temperature of less than 0 °C and a drastic increase in the SCP. After the retreat of the Mongolian-Siberian High in mid-February, the temperature exceeds 0 °C, and the SCP declines rapidly. Therefore, the temperature is closely linked to the SCP in March and May (Table 2), which further suggests that the air temperature is the main factor responsible for the outbreak of snowmelt floods during the spring flood period in Xinjiang (Li & Simonovic, 2002).

From Fig. 12 and Tables 2 and 3, the interannual fluctuations in temperature are generally non-significant between 2001 and 2015. From October to January, although the temperature remains below 0 °C in most cases, the SCP may undergo no change due to sublimated or limited precipitation, which results in low or no correlation between the SCP and temperature. In addition, the annual average temperature in Xinjiang from 2001 and 2015 showed a nonlinear upward trend, and it is not significant. We can indicate that SCP represents the main water resource in arid regions and is closely related to climate change. This phenomenon is more sensitive in arid regions. Therefore, temperature changes will have an important impact on the melting of snow, which will lead to the advancement or delay of floods in spring and summer. Due to the warming, the flood within rivers supplied by snowmelt will be advanced in spring and summer. The correlation between the temperature and SCP strongly supports this view.

## Conclusions

The MOD10A2 and MYD10A2 products from 2001 to 2015 were fused into the MODMYD to better remove partial clouds. Utilizing MODIS images from 2001 to 2015 as well as climate record data, we analysed the temporal and spatial variations in snow cover and snow cover day in Xinjiang and investigated the impact of temperature on the temporal and spatial variations in snow cover.

Most of seasonal snow is concentrated from October to the following April. Snow cover begins to accumulate in October, peaks in January, decreases slightly with rising temperatures in late February and decreases significantly from March to April. During the July-August period, the snow cover is lowest, and only permanent snow on three major mountains remains. In Xinjiang, the average SCD is 75 days, which shows a non-significant decreasing trend from 2001 to 2015. A distinct spatial heterogeneity is noted in the distribution of SCD among various areas of Xinjiang. The SCD exhibits a non-significant decreasing trend in four subregions except for the Ili River Valley. The average SCD is 164 days for the Ili River Valley, 112 days for Northern Xinjiang, 64 days for Southern Xinjiang and 30 days for Eastern Xinjiang. The fluctuation in SCD is most prominent in Eastern Xinjiang, whereas this fluctuation is slight in Southern Xinjiang. High mountains are affected by the dynamics and thermal effects of plateaus, and the SCD of mountains is characterized by a vertical zonal gradient. In the different periods of water and heat in plains and basins, the distribution of SCD is latitudinally/non-latitudinally staggered.

A significant negative correlation is observed between temperature and SCP in spring and winter (*p* < 0.01). Within four subregions, varying underlying land types cause differences in snowfall and snowmelt time occurrences between areas, which results in correlation discrepancies between temperature and month. During winter, the SCAP in Northern Xinjiang and the Ili River Valley reached over 90%. In Southern and Eastern Xinjiang, snow was mainly concentrated in the mountains, whereas in desert region the SCAP is the lowest and ranges between 0 and 30%. In most parts of Xinjiang, a high correlation is found between SCAP and elevation; however, the correlation is low on some high mountain areas, mainly attributed to terrain and other factors. In terms of Xinjiang’s topography, the trend of SCAP at different altitudes is caused by the interaction of the mountain effect and atmospheric circulation.

## Discussion

Although the MOD10A2 and MYD10A2 products were combined, not all clouds can be removed (Xin et al., 2015). The resolution of the MODIS is 500 m, which results in limitations when monitoring alpine terrain due to shadowed pixels (Hall et al., 2002). The forest coverage rate in Xinjiang is 4.87% (Peng et al., 2019), and higher forest coverage occurs in mountainous areas. The accuracy of MODIS snow products in forests could be a little lower than that in the other landscapes (Klein, Hall & Riggs, 1998), and the general albedo trend shows that the glaciers have been darkening since 2000 in the Himalayas (Ming et al., 2015), implying that forest coverage and albedo have a certain influence on the accuracy of snow cover recognition from MODIS. The influences of temperature and precipitation on snow cover variation at different altitudes vary greatly in the Qilian Mountains (Jiang et al., 2016), and those results are consistent with our research.

The NSACI model used in this paper for calculating the relationship between SCAP and elevation is rather simple; however, the relationship between elevation and snow cover is more complex than a linear correlation, and elevation is not the only factor that affects snow distribution (Liu, Ke & Shao, 2015; Pu, Xu & Salomonson, 2007). For instance, we found that in Xinjiang, the terrain (e.g., mountain valley) of some areas has a certain impact on snow cover. However, the application of the NSACI model to the Xinjiang region can reveal the macroscopic relationship between elevation and snow cover in Xinjiang.

Related studies have noted that in past few decades the climate in Xinjiang has changed significantly (Chen et al., 2012). However, Li et al. (2015a) indicated that during the recent warming hiatus period in China from 1998 to 2012, the most notable change was the emergence of a climate pattern from warm dry to warm wet. As temperature is the dominant factor during snowmelt and snow accumulation periods, combining with results of SCP, we found that due to the warming hiatus period, SCP also showed a non-significant downward trend, which also supports the warming hiatus from 1998 to 2012. This change has not only broken the original climate balance but also caused a significant abnormality in the water cycle and atmospheric circulation.

## Additional Information and Declarations

### Data Availability

The following information was supplied regarding data availability: Data is available at NASA’s EarthData. To locate the data: First, go to https://earthdata.nasa.gov/
Second, find the “Search box”, and enter “MOD10A1” or”MYD10A1” to search Third, go to the search result “MODIS/Terra Snow Cover Daily L3 Global 500m SIN Grid V006” and click on it. Fourth, go to the “Search box”, choose a boundary type, such as polygon, rectangle and so on, to determine your download range, and you can see the result of range of you choose in map. Firth, enter “MOD10A1” or “MYD10A1”again in search box; you can see the range you choose already march the “MODIS/Terra Snow Cover Daily L3 Global 500m SIN Grid V006”, and click on it. Sixth, find the different periods for data at the bottom of the web page, you can choose a period, and chick on “Download all”. Seventh, you should Login or register, after login, find the “Select Data Access Method “and choose “Direct Download”; finally, click on “Download data”. Eighth, find the” View/Download Data links” and click on it; you can download the data you need.
